# Gluteal compartment syndrome following drug-induced immobilization: a case report

**DOI:** 10.1186/s13104-015-1003-5

**Published:** 2015-02-08

**Authors:** Andreas Christos Panagiotopoulos, Ioannis Vrachnis, Pantelis Kraniotis, Minos Tyllianakis

**Affiliations:** University of Leicester Medical School, Maurice Shock Building PO Box 138 University Road, Leicester, LE1 9HN UK; Department of Orthopaedics, General University Hospital of Patras, Patras, 26504 Greece; Department of Radiology, General University Hospital of Patras, Patras, 26504 Greece

**Keywords:** Gluteal compartment syndrome, Acute kidney failure, Neuropathic pain, Sciatic nerve palsy, Illicit drug use

## Abstract

**Background:**

Gluteal compartment syndrome is a very rare condition characterized by non-specific symptoms that often lead to misdiagnosis.

**Case presentation:**

We report a case of gluteal compartment syndrome in a 38 year-old Caucasian male (intravenous drug user) following prolonged immobilization due to loss of consciousness. The delay in the appropriate diagnosis and treatment led to a temporary acute kidney injury and to irreversible sciatic nerve palsy.

**Conclusion:**

Delay in the definitive diagnosis and treatment of gluteal compartment syndrome, may lead to higher morbidity of the affected extremity and in rare cases even patient mortality. Special emphasis is given to the aetiology, symptomatology, differential diagnosis as well as the treatment of this condition.

## Background

Compartment syndrome is an orthopedic emergency, which, left untreated, can leads to significant morbidity and even mortality. It is characterized by high intra-compartmental pressures that lead to ischemia of the affected extremity followed by muscle death [1,2].

Most commonly, compartment syndrome develops in the forearm and the lower leg. Gluteal compartment syndrome (GCS) is an extremely rare presentation. The non-specific symptoms often lead to misdiagnosis such as pelvic venous thrombosis [1,2].

The diagnosis is made clinically. Investigations, such as MRI scan, CT scan and ultrasonography are often omitted in order to avoid delays in applying prompt definitive treatment. The gold standard of treatment is urgent fasciotomy. Non-operative treatment is very rarely indicated, only when the patient is unfit for surgery and then the outcome is usually poor [2].

## Case presentation

A 38 year-old Caucasian male presented with a swelling on the lateral and posterior aspects of his left thigh and buttock, complaining also of leg weakness. He admitted using nasally insufflated recreational drugs resulting in loss of consciousness for about eight to ten hours on a staircase. He was aware of the symptoms upon regaining consciousness. His past medical history included chronic intravenous drug use and hepatitis C.

On examination, the lateral aspect of the left thigh was markedly swollen along with the left buttock. They were no signs of redness or skin injury. Left ankle dorsiflexion was weak, with a muscle power grade (MRC) of 3/5, whereas vascular supply was intact. Sensory and motor assessment revealed a left lateral leg deficit indicating left peroneal nerve palsy.

**Figure 1:**
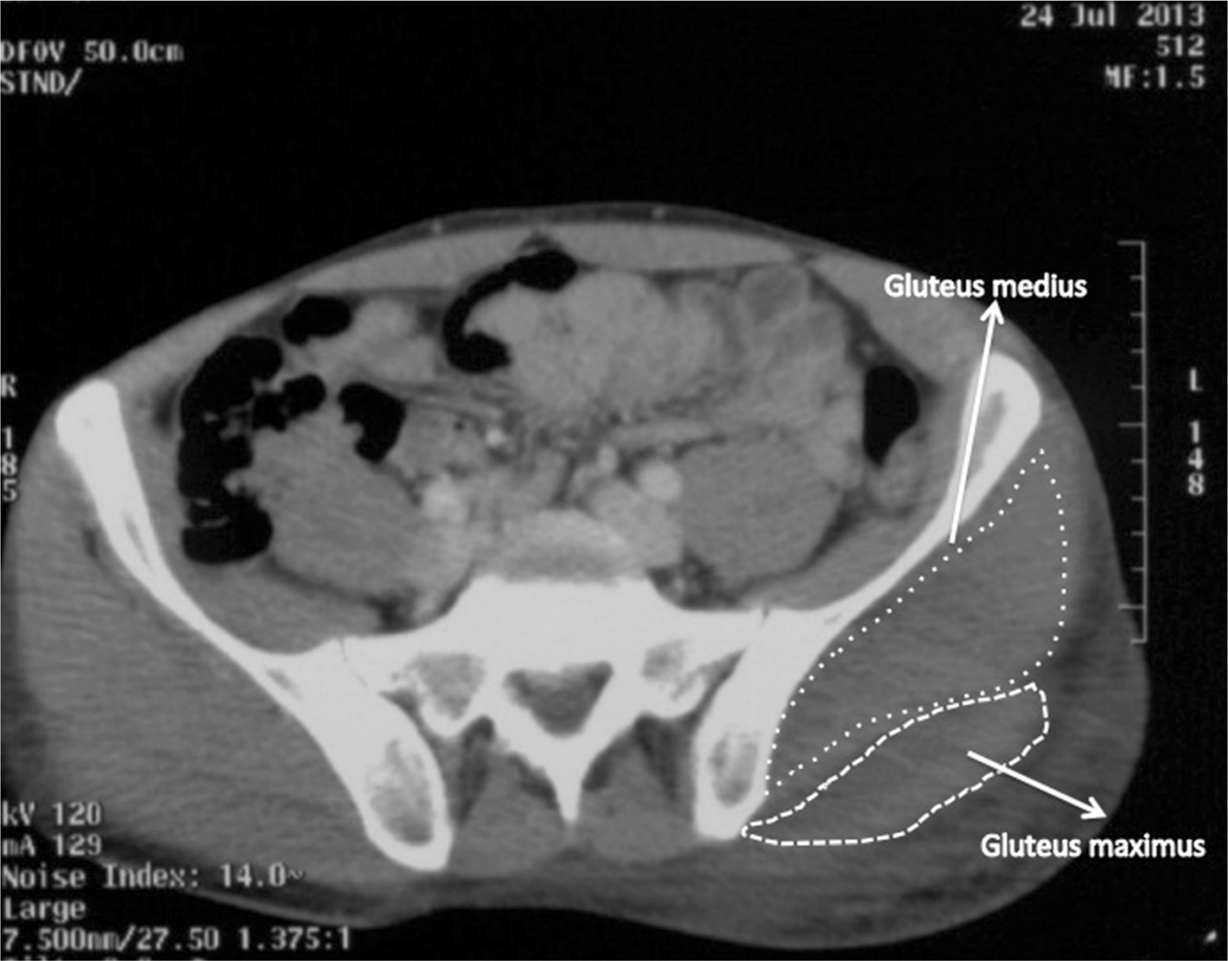
**Transverse CT scan at the level of the iliac crests.** Left gluteus maximus and medius enlargement.

**Figure 2:**
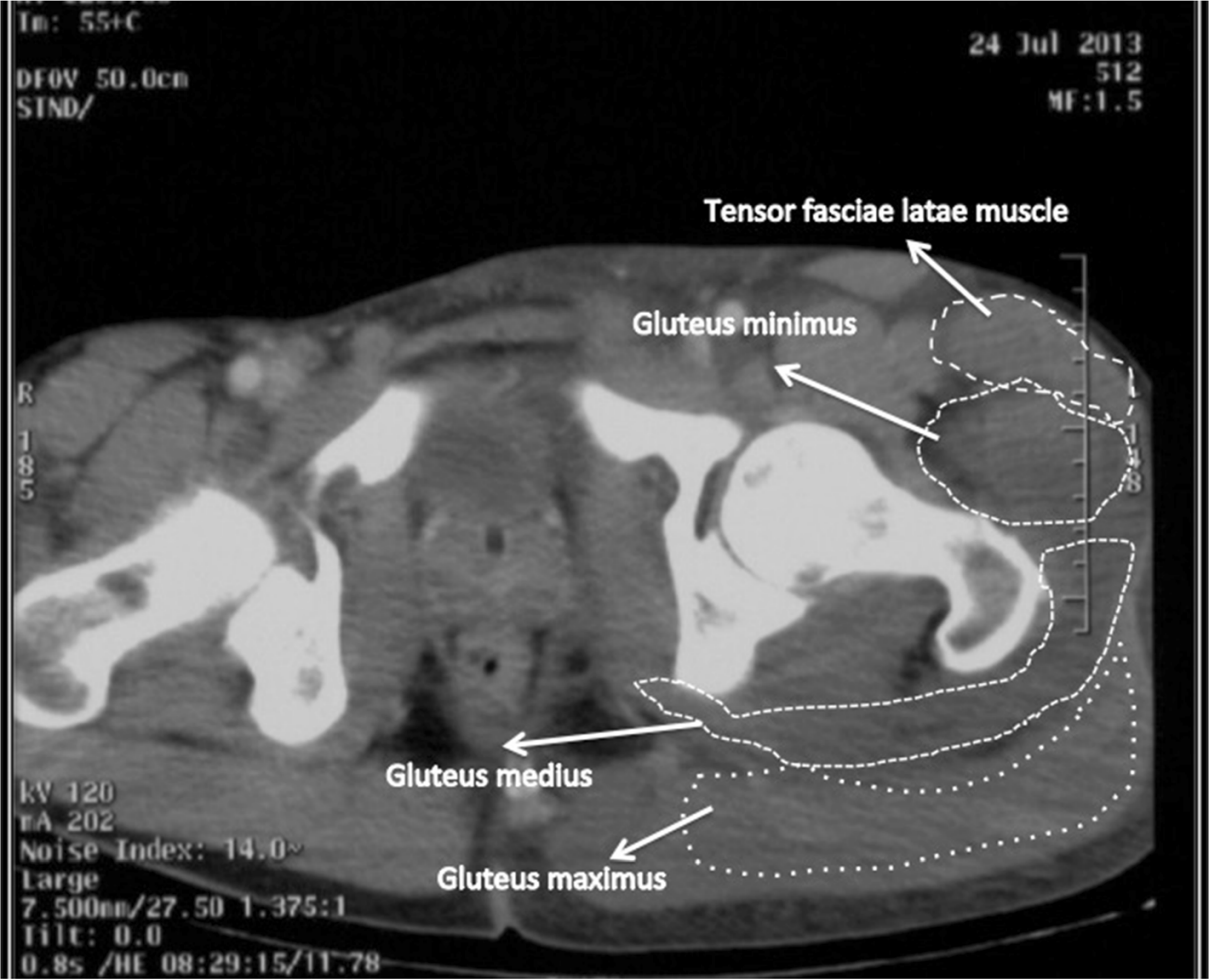
**Transverse CT scan at the level of the femoral neck.** Left gluteal muscles enlargement.

Patient’s biochemistry on admission showed high levels of creatine phosphorkinase (CPK) and lactate dehydrogenase (LDH). Both suggested muscle death and tissue breakdown (Table 1). Imaging with an ultrasound (US) showed non-uniformly swollen left buttock muscle compartments. A CT angiogram was performed to rule out hemorrhage or infection and it revealed signs of muscle and subcutaneous tissues oedema. Moreover the intramuscular fat planes were obliterated. No active arterial or venous bleeding or any abscess was detected. (Figures 1 and 2.)

**Table 1:** **Biochemistry shows high levels of Urea, CPK and LDH**

	**A + E**	**1** ^**st**^ **day**	**2** ^**nd**^ **day**	**3** ^**nd**^ **day**	**5** ^**nd**^ **day**
K^+^ (mmol/l)	-	5.4	4.9	3.7	3.8
Cr (mg/dl)	2.7	2.3	2.6	4.5	6.2
Urea (mg/dl)	66	91	82.3	103	151
CPK (units/litre)	178.000	114.000	77.993	27.156	13.960
LDH (IU/l)	4779	2760	2471	1052	891

Normal values: K^+^ 3.5-5.2 mmol/1, Creatinine 0.9-1.6 mg/dl, Urea 15–54 mg/dl, CPK <190 units/litre, LDH 105–133 IU/L.

Subsequently, the patient became anuric with high levels of urea and creatinine, suggesting acute kidney injury (AKI), which was managed with temporary hemodialysis. Later on, he lost the ability to plantarflex and dorsiflex his left ankle suggesting sciatic nerve palsy. In light of the sciatic nerve palsy and the AKI a diagnosis of GCS made.

He immediately underwent hemodialysis and an urgent fasciotomy to decompress the muscle compartments was performed as soon as the patient was stabilized (12 hours post admission). Post- operatively he required eight units of blood, five units of fresh frozen plasma and five units of platelets. No further hemodialysis was required and the wounds were closed with split skin grafts. Its biochemistry markers (Urea, CPK, Cr, LDH) normalized within the following 15 days and the patient was then discharged. The patient was last seen in the outpatient clinic one year later and the sciatic nerve palsy was still present. He had limited mobility using walking aids and his main complaint was severe neuropathic pain, which was managed with daily Pregabalin intake.

## Discussion

GCS is a rare entity. The leading causes for its development are prolonged immobilization due to alcohol intoxication, illicit drug use and incorrect surgical positioning. Lower limb operations, such as hip or knee arthroplasty, and urological operations are among the most commonly associated procedures [3].

Other more obscure causes include use of anticoagulation and simvastatin medication, intramuscular injections, epidural anesthesia and blunt trauma [3-8].

Patients with GCS can present with severe pain, tenseness and swelling of the buttock as well as numbness and weakness from the sciatic nerve. Moreover on examination, the clinician can report tenderness to touch, hardness of tissues, skin changes, pain on passive motion of the hip joint in any direction, sensory and motor deficits in the sciatic nerve distribution [2]. Out of the two branches of the sciatic nerve, the peroneal (fibular) is more susceptible to injury, thus the patients may present with only isolated foot drop.

However, pain and swelling can also be present in cases of venous thrombosis of the pelvic network, abscess in the thigh or hemorrhage. Venous thrombosis usually affects the pelvic venous network (iliac, superior gluteal, inferior gluteal, internal pudendal, obturator and lateral sacral veins). Differentiation between GCS and venous thrombosis requires measurement of the CPK, the inter-compartmental pressure and imaging of the compartments (ultrasound, MRI and CT scan). Normally the pressure of the muscle compartment is around 13 mmHg but in GCS the pressure rises above 30 mmHg. Imaging studies depict muscle oedema and exclude infection and hemorrhage as causes of the increased intra- compartmental pressure. In case of abscess or hemorrhage, the differentiation with GCS is made using imaging studies (US, MRI, CT scan). CPK and LDH levels are elevated in patients with increased compartmental pressure and their rise is due to muscle death from the hypoxia.

In most cases the diagnosis of GCS is made clinically [9], and because its complications are severe, clinicians should be alerted in order to recognize and manage them promptly. ‘Crush syndrome’ is an umbrella term that describes AKI (Cr and Urea both significantly elevated and myoglobin present in the urine) with hyperkalemia following extensive muscle death.

Hyperkalemia can present either with ECG changes (P wave reduction, peaked T waves, wide QRS complex) or with elevated potassium levels (>5.2 mmol/l). The treatment of crush syndrome is aggressive fluid resuscitation and mannitol alkaline diuresis in order to prevent further kidney damage [10].

A high intra-compartmental pressure is responsible for the initiation of ischemia, hypoxia and necrosis of the muscle and this promotes the release of vasoactive substances, namely vascular endothelial factor (VEGF), endothelin-1 (ET-1) and nitric oxide (NO) [6]. Research has further provided evidence about the low tolerance of the nervous tissue to severe hypoxia and first signs of nerve tissue damage are seen within the first hours. The irreversible changes are seen around the eight-hour mark [10]. In the pelvis, the sciatic nerve runs between external rotators of the femur (Piriformis and the two Gemelli muscles) and the deep parts of the Gluteus maximus. From lateral to medial, there are three gluteal muscle compartments, covered by a thick fascial membrane; first is tensor fascia lata, then the gluteus medius together with gluteus minimus and finally gluteus maximus [3,4,11].

The gold standard treatment of any compartment syndrome is an urgent fasciotomy of the affected compartments. Fasciotomy radically decreases the intra-compartmental pressure thus removing the noxious stimulus of muscle ischaemia, hypoxia and necrosis. Theoretically a fasciotomy is indicated when intra-compartmental pressure measurements are above 30 mmHg for more than 6 hours. In reality fasciotomies are performed when a clinical suspicion is raised from the history and the examination. Beyond twelve hours the outcome is significantly worse [4], whereas early treatment of GCS with an urgent fasciotomy greatly improves the chances for a full recovery of the sciatic nerve [10,12].

Alternatively, non- operative management of a patient with GCS is very rare as the risks are very high and the benefits minimal. Some patients experience complete resolution of their symptoms within 24 months, but others are left with residual incapacitating neuropathic pain and long lasting muscle atrophy [12].

## Conclusion

GCS can develop in individuals that lose consciousness following binge drinking or illicit drug use and remain immobilized for a considerable amount of time at the same position. Inappropriate positioning during lower abdominal, urological and lower limb surgery can also lead to GCS. Urgent fasciotomy is the only viable definitive treatment and it prevents the development of sciatic nerve palsy and acute kidney failure. If there is a delay in decompression, the patient may loose its life and the recovery of the affected sciatic nerve and restoration of function may not be achieved as in the reported case. This highlights the importance of high index of suspicion for proper diagnosis and early treatment of GCS.

## Consent

Written informed consent was obtained from the patient for publication of this Case Report and any accompanying images. A copy of the written consent is available for review by the Editor-in-Chief of this journal.

## Abbreviations

GCS: Gluteal compartment syndrome
AKI: Acute kidney injury
CT: Computerized tomography
MRI: Magnetic resonance imaging
US: Ultrasound
CPK: Creatine phosphorkinase
LDH: Lactate dehydrogenase
VEGF: Vascular endothelial factor
ET-1: Endothelin-1
